# The role of clubfoot training programmes in low- and middle-income countries: a systematic review

**DOI:** 10.1177/0049475520931343

**Published:** 2020-06-23

**Authors:** Sharaf Sheik-Ali, Sergio M Navarro, Evan Keil, Chris Lavy

**Affiliations:** 1 Academic Foundation Doctor, Nuffield Department of Orthopaedics, Rheumatology and Musculoskeletal Sciences, University of Oxford, UK; 2 Surgical Trainee, Department of Surgery, Baylor College of Medicine, Houston, USA; 3 Surgical Trainee, Said Business School, University of Oxford, Oxford, UK; 4 Medical Student, Department of Surgery, 5635 University of Minnesota, Minneapolis, USA; 5 Professor, Nuffield Department of Orthopaedics, Rheumatology and Musculoskeletal Sciences, University of Oxford, UK

**Keywords:** Surgery

## Abstract

While adoption of the Ponseti method has continued gradually, its use to manage patients with congenital talipes equinovarus (CTEV) has been limited in low- and middle-income countries (LMICs) for a number of reasons including a lack of clinical training on technique and lack of appropriate clinical equipment. There are a frequent number of emerging studies that report on the role of clubfoot training programmes; however, little is known in regard to cumulative benefits.

A systematic review was undertaken through Medline, the Cochrane Library and Web of Science for studies analysing clubfoot training programmes. There were no limitations on time, up until the review was commenced on January 2020. The systematic review was registered with PROSPERO as 165657. Ten articles complied with the inclusion criteria and were deemed fit for analysis. Training programmes lasted an average of 2–3 days. There was a reported increase in knowledge of applying the Ponseti method in managing clubfoot by participants (four studies *P* < 0.05). Skill retention was examined by multiple choice (MCQ) examination style questions before and after the training programme in two studies; both showed an improvement (MCQ answers improved from 59% to 73%). All studies showed an improvement in participants' self-reported understanding of the Ponseti method and confidence in its use in future practice (*P* < 0.05). There were improved benefits of knowledge and clinical application of the Ponseti method by participants in the programmes in all studies examined. However, there was a significant lack of follow-up and exploration of long-term effects of these programmes. Implementing training programmes based on perceived benefits rather than actual long-term benefits may have a negative impact on healthcare delivery and patient management in LMICs.

## Introduction

Congenital talipes equinovarus (CTEV) is one of the most prevalent congenital deformities, estimated to occur in 0.6–1.5 per 1000 live births, with 80% born in low- and middle-income countries (LMICs).^1^ It is defined as fixation of the foot in adduction, varus and in supination, rotated outwards and downwards.^2^

The advent of the Ponseti method to manage CTEV has dramatically improved patient outcomes when compared to the invasive surgical approach.^3–7^ The method consists of two key elements: first, correction of the deformity by manipulation and serial casting; and second, the use of abduction braces to maintain achieved correction.^8,9^ While adoption has continued gradually, the use of the Ponseti method to manage patients with CTEV has been limited in LMICs for a number of reasons: a lack of clinical training on the technique and a lack of appropriate clinical equipment.^10^ Reasons for this may include limited training in the Ponseti method, limited personnel and competing priorities, particularly in sub-Saharan Africa.^10,11^ Further, the major challenge for the availability of surgical services for children in LMICs worldwide is low-skilled workforce and infrastructure.^12–14^ A recent Zambian study, using the World Health Organization (WHO) SAT tool, also demonstrated that lack of surgical skill and relevant equipment was the leading factor in limiting availability of 93% of paediatric procedures.^15^

Training programmes and short-term courses appear beneficial in terms of addressing the issue of lack of clinical skills.^16^ These typically consist of ‘hands-on workshops’ and theoretical lectures on the Ponseti method that span over 1–7 days.

Given the expenditure and burden of running potentially expensive courses in LMICs which might have otherwise prioritised their available funds/time, training programmes have an ethical responsibility to clarify benefit against costs. As such, the importance of an evidence-based approach is vital to consider. Costs of running the programmes and long-term retention/application of skills are factors that should be monitored closely with such programmes.

The aim of the present article is to systematically review the literature to determine the role and long-term effectiveness of clubfoot training programmes in LMICs and categorise these reported benefits. In the event that statistical reported benefits differ, we shall use a narrative analysis to identify common themes and discuss in depth the reported benefits and significance of them.

## Methods

A systematic review was undertaken through Medline, the Cochrane Library and Web of Science. There were no limitations on time, up until the review was commenced in January 2020. The systematic review was registered with PROSPERO as 165657.

A literature search was conducted electronically with no time constraints using ‘(“Congenital equinovarus” or “CTEV” or “Clubfoot”)’ AND ‘(Programme)’, including MeSH terms (training programmes, teaching) with no language restrictions. Included were the introduction of training programmes specific to management of clubfoot in LMICs; excluded were training programmes not related to clubfoot and where no clear description of training programme implemented was given.

**Figure 1.**
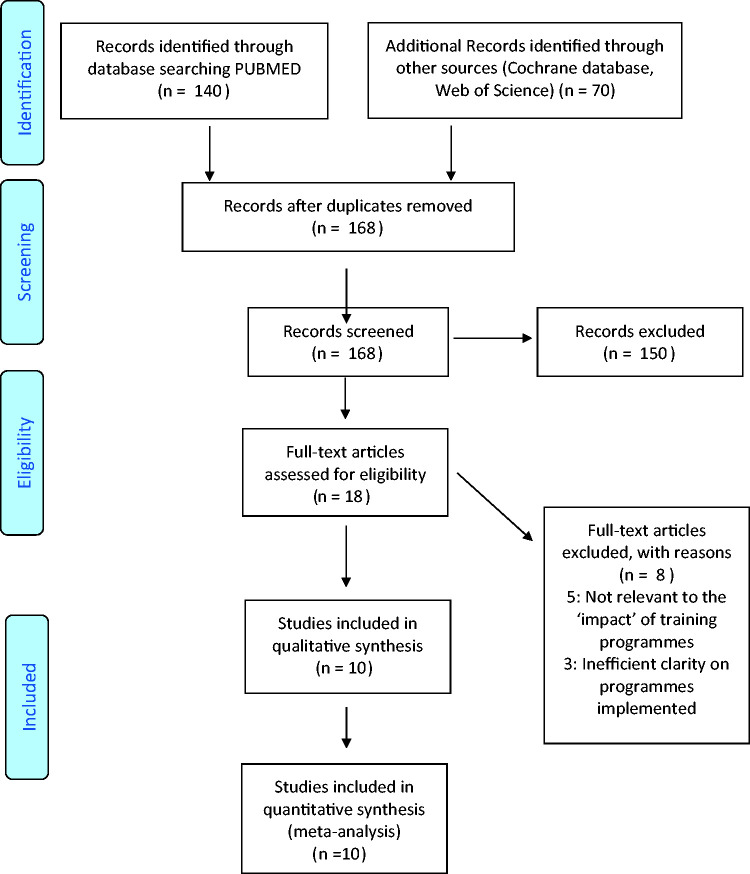
PRISMA flow diagram.

Following the recommended PRISMA guidance, three authors (SS, SN and EK) examined the selected studies against an inclusion criterion (Figure 1). Inclusions were specific to clubfoot or congenital equinovarus management training programmes. We did not exclude articles based on chosen technique (e.g. Ponseti or other manipulative methods). A secondary search was undertaken, which involved screening references from selected texts for further studies. Duplicates were removed and any disputes noted between the two authors on eligibility of studies were discussed with the third author.

Key data including author names, date of publication, study design, location, number of participants, duration of follow-up and results were extracted and formulated. We included a quality analysis for quantitative studies for further evaluation, including risk of bias assessment.

Where possible, a data analytical analysis was performed for improvement in skills, confidence after training and retention of skills gained. If different statistical methods were used, a narrative analysis on common themes was opted for.

The Cochrane tool for assessing risk of bias was used to assess the quality of included studies, adapted from Higgins and Altman.^17^

## Results

Ten articles complied with the inclusion criteria and were deemed fit for analysis. All papers reviewed were published in 2007–2018.

There was a reported increase in the knowledge of the Ponseti method in managing clubfoot in participants in all studies. Two studies opted for multiple choice (MCQ) examination style questions before and after the training programme; both showed an improvement (MCQ answers improved from 59% to 73%). All other studies opted for the use of questionnaires that were given after the training programmes were completed (range = 1–5 days), focusing on confidence and perceived improvement in understanding of the Ponseti method after the training session. All studies showed an improvement in participants' self-reported understanding of the Ponseti method and confidence in its use in future practice (*P* < 0.05).

Four studies offered data on follow-up of programmes (Table 3, supplementary index). Owen et al.^18^ was the only study to show an increase in the numbers of providers, patients treated and centres that provide the Ponseti method of clubfoot management over two years after implementation of the training programme: from 57 new Ponseti providers immediately after the training programme to 339 in over two years.^18^ Lavy et al.^19^ showed that in one year, 20/25 newly formed Ponseti clinics were still active and had seen >342 patients during that time period. There was a significant lack of data from the majority of studies on long-term follow-up.

**Table 3.** Reported results of number of trained providers, patients treated and treatment centres established resulted from providers completing CTEV training courses.

	Providers (n)			Treatment (number of patients)			Number of centres		
Total number	Immediate	1 year	2 years	Immediate	1 year	2 years	Immediate	1 year	2 years
Owen et al.^18^ (2012)	57	238	339	N/A	1256	2645	28	49	33
Lavy et al.^19^ (2007)	307	307	N/A	N/A	342	N/A	25	20	N/A
Radler et al.^36^ (2018)	7	7	7	105	N/A	52	1	1	1
Nogueira et al.^20^ (2011)	489	434	N/A	N/A	3469	N/A	N/A	N/A	N/A
Evans et al.^34^ (2010)	67	67	67	11	30	N/A	N/A	N/A	N/A

Nogueira et al.^20^ showed the most significance in knowledge gained by participants after the training programme/symposium, going from 17% to 88% self-reporting that they were able to treat children with the Ponseti technique (*P* < 0.05). Of them, 94% attributed this to the training programme.

Training programmes lasted an average of 2–3 days in the studies examined. The content of the training programmes varied between hands-on casting, lectures/workshops and directly observing qualified Ponseti clinicians manage patients.

To analyse gained benefits, the majority of studies utilised questionnaires before and after the programme. The rate of completion for the questionnaires was in the range of 50%–70%. The duration for completion was in the range of 1–5 days after the programme. Few studies examined the benefits of programmes in relation to skill retention after seven days.

The majority of participants in the programmes were orthopaedic surgeons, physiotherapists and nurse practitioners. No study examined the differences between skills retained and practised between non-physicians and physicians.

There were no randomised control trials/comparative studies found.

### Quality analysis

Most studies reviewed were observational. Overall, the majority of studies were reported as fair, with lack of reporting on withdrawals/drop-outs reducing a large proportion of the quality of the studies. Further, only few studies reported yearly assessments. Most studies scored 2–3 for methods of data collection, reflecting on the frequent use of self-reported questionnaires and rarely any objective assessment of retained skill.

## Discussion

On review of the available literature on CTEV training programmes in LMICs, we have identified common themes of improvement in knowledge of management of CTEV and a subsequent increase in the proportion of clinical cases managed by attendees in their own practice (Tables 1 and 2). Most studies consisted of 2–3-day programmes that focused on casting and monitoring for relapse. An attribute to training programmes that is difficult to quantify is the follow-through of subsequent benefits when trained participants in LMICs pass on gained knowledge to their co-workers and place of practice. From a patient perspective, the application of the Ponseti method instead of invasive surgery provides reduced healthcare costs and improved outcomes.^21^ However, there was some disparity on the contents of each clubfoot training programme.

**Table 1.** Description of the studies included in the present review.

Results: studies	Study design	Location	Participants (n)	Participants	Training timeline	Results	Follow-up	Quality of study – global rating
Smythe et al.^31^ (2018)	Prospective cross-sectional study (pre + post questionnaires	Multiple (Ethiopia, Rwanda, Kenya)	164	Regional trainers, treatment experts, national clubfoot treatment providers	4-day training programme	After programme: MCQ answers improved from 59% to 73%. Self-reported confidence increased from 57% to 89%	Immediate	Fair
Smythe et al.^31^ (2018)	Mixed-methods study					Post programme: mean participant confidence increased from 64% (95% CI: 59–69) to 88% (95% CI: 86–91) post course. Mean participant knowledge increased from 55% (95% CI: 51–60) to 78% (95% CI: 76–81).		Good
Vaca et al.^32^ (2018)	Prospective survey study	Tanzania	30	Orthopaedic surgeon, general practitioner, physical therapist, occupational therapist, nurse and clinical officer	2-day training programme supplemented by online e-learning	Post-programme: participant knowledge improved on scoring from 44 ± 12.5 to 69.8 ± 16.5 (*P* < 0.0001) and 44.3 ± 14.0–67.9 ± 21.4 (*P* = 0.01), respectively. 100% of participants expressed being comfortable with training design and delivery	Immediate	Fair
Asitha et al.^33^	Mixed-methods retrospective survey study	Multiple (USA and LMICs)	131	Orthopaedic surgeons, physical therapists, nurse practitioners	2-day onsite training program	At follow-up: 97% percent of practitioners reported currently using the Ponseti method as the preferred treatment for clubfoot, with a satisfaction rate of 97% by participants	1–10 years	Fair
Evans et al.^34^ (2009 )	Prospective questionnaire study	Vietnam	36	Healthcare providers, physical therapist, nurses and surgeons	3-day training programme	At follow-up: 93% (23/25) using the Ponseti method follow-up guidelines to monitor for clubfoot relapses. 97% (31/32) trainees were disseminating the Ponseti technique to colleagues within their institutions	Unable to be determined	Good
Owen et al.^18^ (2012)	Observational data analytical study	Multiple (10 LMICs)	634	Orthopaedic surgeons, orthopaedic technicians or clinical officers, physical therapists and prosthetists/orthotists	Unspecified-length training course	Multi-year follow up: at one year, 110 clinics established and functioning, 7705 children enrolled for the study.	1, 2 and 3 years	Good
Nogueira et al.^20^ (2011)	Questionnaire-based analytical study	Brazil	556	Orthopaedic surgeons	2-day training course	Post-programme and 1-year follow-up (only 4% responded to survey) Questionnaire before and after study at 1 year; 17% did not know of the Ponseti method before the teaching. 88% felt they were able to treat children with the Ponseti technique after the symposium. 94% of respondents reported that the symposium changed their way of treating clubfoot.	1 year	Fair
Lavy et al.^19^ (2007)	Prospective cohort study	Malawi	307	Orthopaedic clinical officers, nurses, medical assistants	3-day training course	1-year follow-up: led to the establishment of a regional clubfoot clinic. Twenty out of the 25 clinics originally established were still active, and over one year had seen a total of 342 patients	1 year	Good
Radler et al.^35^ (2010)	Cohort study	Mali	7	Healthcare workers	Two unspecified-length training courses	At follow-up: 40/52 patients with ‘good’ or ‘medium’ outcomes after two years of treatment with the Ponseti method	2 years	Fair
Locke et al.^36^ (2019)	Qualitative study	Multiple (Madagascar, UK)	18	8 doctors from 6 different centres and 10 physiotherapists began the course	400 h of onsite teaching courses	Post programme: OSCE taken by participants after completion. All 8 physicians successfully completed the course, 7/10 physiotherapists completed the course	Immediate	Fair

CI, confidence interval; LMIC, low- and middle-income country; MCQ, multiple choice question; OSCE, Objective Clinical Situation Evaluation.

**Table 2.** Cochrane's tool for assessing risk of bias (adapted from Higgins and Altman^18^).

Studies	Selection bias	Study design	Blinding	Data collection methods	Withdrawals + drop-outs	Intervention integrity	Analysis	Overall score	
Smythe et al.^21^ (2018)	1	1	3	2	None reported	1	1	2	Fair
Vaca et al.^32^ (2018)	2	2	3	2	None reported	1	1	2	Fair
Smythe et al.^31^ (2018)	1	1	3	1	Partial response	1	1	1	Good
Asitha et al.^33^ (2013)	2	2	3	2	None reported	1	1	2	Fair
Evans et al.^34^ (2010)	2	2	3	3	N/A	2	3	2	Fair
Owen et al.^18^ (2012)	2	1	3	1	Partial response recorded	1	1	1	Good
Nogueira et al.^20^ (2011)	2	2	3	2	Low response rate	2	2	2	Fair
Lavy et al.^19^ (2007)	1	1	3	1	5/25 centres	1	1	1	Good
Radler et al.^35^ (2018)	1	2	3	3	<50% retention rate	1	1	2	Fair
Locke et al.^36^ (2019)	1	2	3	1	N/A	1	2	2	Fair

Scoring is as follows: 1 = strong; 2 = moderate; 3 = weak.

N/A, not applicable.

Given the prevalence of CTEV, particularly in LMICs,^1^ non-invasive methods of managing the condition are a significant cost-efficient technique in comparison to invasive intervention. Thus, short training programmes to teach LMIC participants the Ponseti method as well as other non-invasive methods to implement in their own practice are well warranted.

Furthermore, on quantifying the economic burden of clubfoot in LMICs, we note a reported average of 7.42 disability-adjusted life years (DALY) averted with a cost-effectiveness ratio of US $22.46 per DALY averted in sub-Saharan African countries as a result of contribution to society due to CTEV.^22^ Other studies have reported similar findings.^23^ Considering the low costs of training programmes in relation to DALY, it would make financial sense for LMICs to continue invest in training methods of non-invasive techniques for its healthcare delivery personnel.

However, training programmes reported in the literature do not provide a reliable measure of long-term impact given the short duration of follow-up. Previous studies have alluded to loss of knowledge gained unless continually used or reassessed in a given capacity.^24^ The authors' suspicions are that although training programmes may be beneficial in the short term, it would be wise to reassess gained skills to cement the notion that clubfoot training programmes benefit LMICs, as well as ensure the correct level of quality of care provided by the trained participants.

Reasons for the lack of follow-up may include the relatively new introduction of CTEV programmes in LMICs, difficulties in following up a programme due to change of personnel/loss of contact, and lack of sufficient programme funding to allow further studies to evaluate outcomes.^10,25^ Programmes that did record follow-ups demonstrated some coherent structure to evaluate the benefits of implemented programmes over a recorded timescale (Table 3).

Follow-up on implementation of learnt skills would require well-recorded analytical data on patients seen and treated by personnel who attended the training programmes. These data would need to be collected objectively. There is a general lack of quality-assured hospital data and audit in hospitals in LMICs that have been previously explored in prior studies. This could suggest one of a number of possible reasons behind the lack of follow-up.^26^

Relapse rates of CTEV have been associated with lack of parental guardians' knowledge on Ponseti casting and the importance of follow-up clinics.^27^ It is therefore of some surprise that not all training programmes in the literature fostered the importance of parental education as a skill to their trainees. Inclusion of educative methods for families provided by clinicians should be intertwined in training programmes.^27^

Content of training programmes varied, mainly in the personnel taught, ranging from physiotherapists and doctors to non-physician clinical officers. The majority of the programmes included lecture-based study with practical elements. Arguments have been made for a uniformed, established, reproducible training curriculum for manipulative methods on managing clubfoot in LMICs.^21^ A good example is the Africa Clubfoot Training Project, part of the Global Clubfoot Initiative, a project that reinforced a standardised curriculum of clubfoot training in Ethiopia and aligned with local priorities through joint planning and coordination. However, data on follow-up and the impact of said training are yet to be made available.

A well-managed training programme must take into consideration the materials that are sourced in LMICs, the number of cases of clubfoot seen by each practitioner, and follow-up on the effectiveness of the programme to monitor the following:

1. Are skills still being practised and retained?
2. Knowledge still present.
3. Clinics still functioning.
4. Issues identified as a result of the training and managed appropriately (audit and quality improvement).

The importance of the evidence base for medical/surgical training has been well explored.^28,29^ Implementing training programmes based on perceived benefits rather than actual benefits may have a negative impact on healthcare delivery and management of patients in LMICs. It is thus incredibly important that alongside clear rationale, which the majority of CTEV training programme studies provide, there must be a structured, robust follow-up scheme in place to employ good sustainability of reported benefits. Neglecting the long-term effects of training programmes can cause under-reporting of complications and overestimation of perceived benefits.^30^ Further, important factors that must be objectively explored include the level of skill retention over a longer duration and continuous assessment of participants current/future practice. The bias in self-reported questionnaires may not report the full extent of the impact of training. Rather, we suggest objective assessments such as MCQs or Objective Clinical Situation Evaluations (OSCEs) as a formal assessment of retained skills. These should ideally take place over a yearly time span.

## Conclusion

The present study highlights the effects of CTEV training programmes in LMICs. There are improved benefits of knowledge and application in all studies examined. However, there is a significant lack of follow-up and long-term effects of said programmes. As a result, it is difficult to ascertain the long-term benefits of implementing these programmes. The authors agree that this can be ameliorated with objective follow-up measures in place to examine application of knowledge and skill. Given the number of studies that highlight reasons for relapse of CTEV, training programmes would do well to address highlighted reasons such as lack of parental understanding of the condition, as well as foster long-term collaboration and partnership between institutions to ensure long-term follow-up from training programmes. Future studies should focus on the long-term effects of CTEV training programmes on the impact on practice in LMICs.

### Strengths and limitations

This is the first systematic review in the literature assessing the impact of CTEV training programmes in LMICs. A robust analysis of the literature revealed a significant lack of important follow-up data on CTEV training programmes.

The limitations of this study include the small sample size and lack of current CTEV training programme studies. This could be due to the recent implementation of such programmes in LMICs.
